# Green Synthesis of Titanium Dioxide Nanoparticles: Physicochemical Characterization and Applications: A Review

**DOI:** 10.3390/ijms26125454

**Published:** 2025-06-06

**Authors:** Nasir Shakeel, Ireneusz Piwoński, Parvaz Iqbal, Aneta Kisielewska

**Affiliations:** 1 Department of Materials Technology and Chemistry, Faculty of Chemistry, University of Łódź, Pomorska 163, 90-236 Lodz, Poland; aneta.kisielewska@chemia.uni.lodz.pl; 2 Doctoral School of Exact and Natural Sciences, University of Łódź, Jana Matejki 21/23, 90-237 Lodz, Poland; 3 Government Graduate College, Chowk Azam, Layyah, Multan 31-450, Pakistan; parvaziqbal85@gmail.com

**Keywords:** eco-friendly remediation, pollutant degradation, sustainable environmental solutions, green chemistry

## Abstract

Nanotechnology is an emerging field in science that exhibits significant promise in the synthesis of nanomaterials for diverse applications. Traditionally, these nanomaterials were manufactured using hazardous and labor-intensive physical and chemical processes. Nevertheless, in recent years, researchers have developed safer, more scalable, and environmentally friendly methods for green synthesis. The problem addressed in this study is the need for an environmentally friendly and efficient synthesis process for titanium dioxide nanoparticles (TiO_2_ NPs) with enhanced properties. The aim of this work is to describe the synthesis of TiO_2_ NPs with various plant extracts using a green approach and to evaluate the physicochemical characteristics and potential applications of the resulting nanoparticles. This study focuses on understanding how the integration of plant extracts influences the properties of TiO_2_ NPs, particularly in terms of their structural, optical, and functional characteristics. The novelty lies in the use of plant extracts as bio-reductants and capping agents, which not only provides a safer and more sustainable synthesis method but also enhances the functional properties of TiO_2_ NPs. This green synthesis approach reduces the use of harmful chemicals, making the process more environmentally friendly and economically viable, with potential applications in photocatalysis, antibacterial, and antioxidant activities. The TiO_2_ NPs possess diverse functionalities, including photocatalysis, antibacterial properties, and antioxidant properties. The initial precursor, such as a metal salt, undergoes transformation into the desired nanoparticles through the actions of plants exactly. Bio-reduction and capping processes are carried out by secondary metabolites found in bacteria and plants. The results demonstrated that the plant extract-mediated TiO_2_ NPs exhibited enhanced photocatalytic activity, superior antibacterial effects, and higher antioxidant potential compared to chemically synthesized TiO_2_ NPs. This highlights the potential of green synthesis methods in producing nanomaterials with improved functional properties for a wide range of applications.

## 1. Introduction

**Figure 1:**
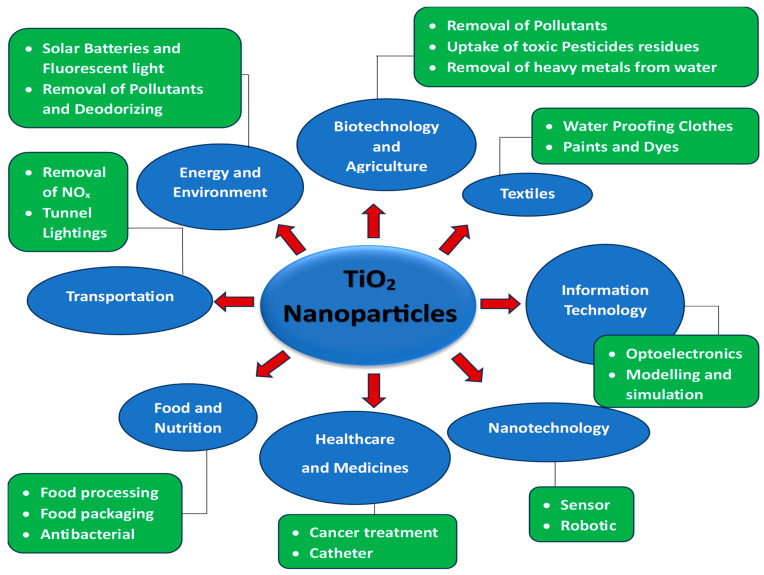
Potential applications of TiO_2_ nanoparticles in various fields of life.

Global environmental degradation and an energy dilemma are currently endangering humanity’s long-term steady growth. Seeking high-efficiency and environmentally friendly protocols to address environmental pollution and energy constraint challenges has become an urgent endeavor [1,2]. It is critical for researchers to develop efficient, stable, and green solutions to successfully regulate environmental pollution and tackle energy concerns [3]. Figure 1 shows the potential applications of TiO_2_ nanoparticles in various fields of life.

Fujishima and Honda [4] were the first to demonstrate photocatalytic water splitting on a TiO_2_ electrode in 1972. Their work advances semiconductor photocatalysis, which is widely employed in the fields of environment and energy, opening the door to photocatalysis and attracting the attention of many scientific scholars. In recent years, the research and development of nano-TiO_2_ (nano-TiO_2_ commonly refers to particles smaller than 100 nm in at least one dimension) [5,6,7] photocatalysts has progressed rapidly, with increasing accomplishments. TiO_2_, one of the most researched photocatalysts, can only absorb UV light due to its relatively high band gap of 3.0–3.2 eV, which reduces photocatalysis efficiency [8,9].

It is intended to decompose waste products efficiently under UV light irradiation due to its band gap above 3.0 eV. However, due to its broad band gap, visible light activity is often hampered, and it is known that solar energy contains around 4–5% UV light and 50% visible light. As a result, with so little UV light reaching the earth’s surface, the usage of UV-active photocatalytic materials is not economically possible [10].

TiO_2_ mixed with metal NPs has been researched to overcome this problem, where the metal NPs enhance the catalytic capabilities of TiO_2_ by tuning the Fermi level of TiO_2_ and acting as an electron sink [11]. Various ways for changing their properties have been developed, including the formation of nanoparticles by the addition of other nanomaterials.

Many remarkable investigations have been conducted on the synthesis and modification of TiO_2_-based photocatalysts, as well as their applications in alleviating energy and environmental challenges [12,13,14]. However, few papers have classified the environmental uses of nano-TiO_2_ and compared the specific applications of nano-TiO_2_-based photocatalysts [15,16,17]. Phytochemical doping, surface state modifications, and heterojunction formation are some of the mechanisms that contribute to the significantly lower band gaps seen in plant-mediated TiO_2_ nanoparticles (NPs) when compared to the typical anatase phase (~3.2 eV), rather than just quantum size effects. Phytochemicals such as flavonoids and polyphenols can replace oxygen sites in TiO_2_ by acting as sources of carbon (C) or nitrogen (N). For instance, the hybridization of N 2p and O 2p orbitals results in a smaller band gap in N-doped TiO_2_ [18,19]. Extracts such as Taraxacum officinale include functional groups (-OH, -COOH) that stabilize oxygen vacancies (Vo), which lower the effective band gap by introducing defect states below the conduction band [20]. By increasing interfacial interactions between TiO_2_ and secondary phases (such as carbon coatings), phytochemicals such as inulin from dandelion roots reduce recombination and alter band edges [21]. As demonstrated in Ag-doped TiO_2_ NPs, where plasmonic effects predominate, plant-mediated synthesis frequently counteracts the effects of quantum confinement, which normally increases band gaps in ultrasmall NPs (<5 nm), by inducing surface states that are mitigated by phytochemicals [22]. The band gap of TiO_2_ was modified to improve its photocatalytic activity by doping it with metal and nonmetal ions, introducing vacancy, and constructing composites with other semiconductors [23].

**Figure 2:**
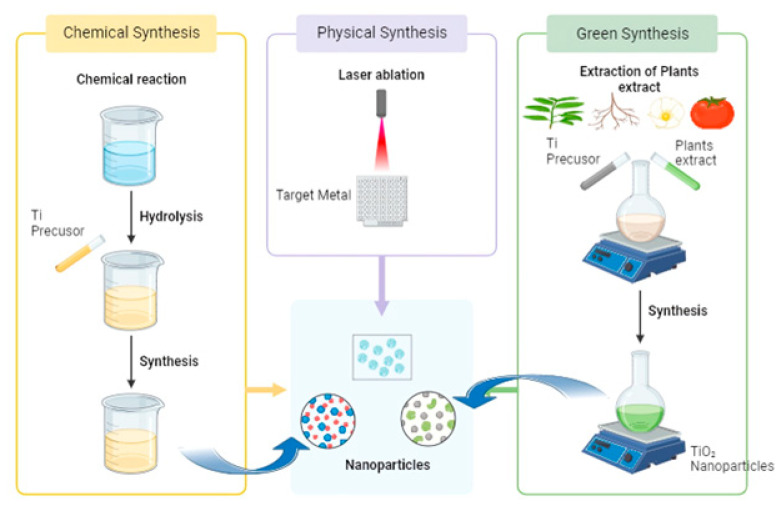
Synthesis approaches, such as chemical, physical, and green methods.

Various synthesis approaches, such as chemical, physical, and green methods, have been utilized in the manufacture of NPs (as shown in Figure 2). These approaches are now encountering issues such as aggregation, stability, crystal growth control, size, shape, and distribution due to their ongoing development stage. In addition, the separation of the generated NPs is a crucial factor to be considered for future applications. The use of bacteria, actinomycetes, yeasts, fungi, algae, and plant extracts in green synthesis provides a superior alternative to chemical and physical methods in the development of green technology. Furthermore, it has the capability to avoid the utilization of dangerous chemicals, is cost-effective and environmentally friendly, and can be easily expanded for large-scale synthesis [24]. It is more energy- efficient without the need for high pressure and temperature [25]. While chemical and physical procedures are commonly employed for the manufacture of NPs, they necessitate the utilization of reactive and poisonous reducing agents [26].

Efforts are ongoing to establish a straightforward, dependable, and efficient method for producing NPs through environmentally friendly synthesis processes. The collective endeavors of all researchers are crucial in generating stable, highly reliable, and well-functionalized NPs. Hence, it is imperative to investigate a sustainable methodology to produce NPs. The concern may encompass the economic viability, environmental sustainability, social adaptation, and availability of local resources. Green synthesis methods commonly involve natural, sustainable materials such as plant extracts, microorganisms, or other biocompatible substances. This method decreases the utilization of dangerous chemicals and solvents linked to conventional industrial techniques, leading to a decreased environmental footprint. Nanoparticles of TiO_2_ synthesized through environmentally friendly techniques exhibit greater biocompatibility in comparison to those manufactured using traditional chemical methods.

Although the goal of both green and chemical synthesis methods is to create TiO_2_ nanoparticles, these approaches vary significantly in terms of their environmental impact, safety, scalability, and the characteristics of the materials and processes used. Green synthesis methods are preferred due to their sustainability and potential for biocompatibility, which makes them more appealing for applications that priorities eco-friendliness and safety. When TiO_2_ nanoparticles are produced through environmentally friendly methods (such as using plant extracts or microorganisms) the resulting nanoparticles maintain their original chemical properties. The selection of the synthesis technique can impact distinct attributes such as particle dimensions, structure, and surface characteristics. However, the fundamental chemical properties of TiO_2_ remain constant regardless of the chosen synthesis approach.

The purity and crystalline phase of TiO_2_ NPs produced through environmentally friendly methods may differ based on the synthesis conditions and parameters. The purity of the resulting TiO_2_ NPs can be affected by the purity of the starting materials, such as the titanium precursor, and the efficiency of the green synthesis process in reducing impurities. By manipulating synthesis parameters such as the reaction temperature, precursor concentration, pH of the reaction mixture, and nature of the green reducing or stabilizing agents, one can regulate the crystalline phase of TiO_2_ NPs, including anatase, rutile, or mixed phases. Phytochemicals (such as flavonoids, polyphenols, and -OH and -COOH groups) found in plant extracts serve as capping and reducing agents. These biomolecules regulate growth and nucleation to stabilize stages. Because of their strong chelation of Ti^4+^ surface sites, which slows hydrolysis and allows for ordered atomic organization, extracts high in polyphenols (such as Tinospora cordifolia [27] and Impatiens rothii [28]) favor anatase. By changing surface energy or promoting phase transitions at higher temperatures, amine-rich extracts may encourage rutile [29]. Because of kinetic control, green technologies frequently generate anatase at moderate temperatures (e.g., 50–150 °C) [30]. Phase transitions between anatase and rutile are triggered by post-synthesis heating (>500 °C). For instance, mixed anatase–rutile phases with increased photocatalytic activity were observed in TiO_2_ NPs calcined at 500 °C [31].

Titanium dioxide is categorized as a substance with a moderate suspicion of causing cancer through inhalation (Category 2) according to the Classification, Labelling, and Packaging (CLP) Regulation. Regulation (CLP) (EC) No. 1272/2008 imposes precise labeling obligations for products containing TiO_2_, which include the use of hazard pictograms, signal words, and precautionary statements. The disparity in waste generation between green and industrial TiO_2_ synthesis methods primarily stems from the sustainability and environmental factors associated with the respective processes. Green synthesis methods aim to reduce waste and environmental impact by utilizing renewable resources and eco-friendly processes. In contrast, industrial methods can produce more waste and have a larger environmental footprint due to their larger scale and reliance on traditional chemical processes. Utilizing green synthesis methods can thus aid in achieving a more sustainable and environmentally conscientious production of TiO_2_. Green synthesis decreases expenses by utilizing plant extracts, frequently derived from agricultural waste, while minimizing dangerous chemicals and reducing energy demands relative to conventional processes. Agricultural by-products are utilized for nanoparticle manufacturing, hence minimizing waste. The procedure mitigates environmental effects by recycling natural resources and reducing toxic chemicals, promoting long-term sustainability and supporting a circular economy [32,33,34,35]. The circular economy aspect of chemical and specific green synthesis (based on plant extracts like *Rhizophora apiculata*, *Ocimum sanctum* and *Butea monosperma*) is shown in Table 1.

Waste containing TiO_2_ must be handled and disposed of in compliance with the Waste Framework Directive (WFD) (Directive 2008/98/EC) to minimize environmental effects and ensure appropriate waste management and recycling. The energy balance in the green synthesis of TiO_2_ entails a comprehensive assessment of energy inputs, process efficiency, the utilization of renewable energy, and environmental impacts to guarantee the sustainability and eco-friendliness of production methods. Prioritizing the reduction of energy consumption and the utilization of renewable resources are crucial in green synthesis methods for TiO_2_ NPs.

However, numerous obstacles persist in the eco-friendly production of TiO_2_ NPs. A primary problem is the reproducibility of results, as discrepancies in plant species, cultivation conditions, and extraction techniques might result in inconsistencies in nanoparticle size and functioning [36]. By multipurpose interactions that are absent in traditional chemical synthesis, plant-derived capping agents (such as polyphenols, -COOH, and -OH groups) improve the durability and reusability of TiO_2_ nanoparticles (NPs). By physically adhering to TiO_2_ surfaces, large biomolecules (such as proteins and polysaccharides) form a dense organic shell that inhibits aggregation through steric hindrance. Aloe vera polysaccharides, for instance, envelop NPs in a hydrogel-like layer that defies van der Waals forces [37]. Undercoordinated Ti^3+^ surface sites are saturated by plant biomolecules (such as flavonoids and tannins) that chelate Ti atoms via –OH/–COOH groups. This preserves NP integrity during reuse by lowering oxidative degradation and ROS production (such as ^•^OH radicals) [38]. By scavenging free radicals, polyphenols (such as quercetin and catechins) protect TiO_2_ surfaces from photocorrosion. This defense is absent from chemically produced NPs, which break down more quickly in reactive settings [39]. Moreover, expanding the green synthesis technique for industrial use while preserving its ecological advantages presents considerable technical challenges. The precise mechanisms by which plant metabolites promote nanoparticle production are poorly understood, complicating optimization efforts.

Despite these challenges, the prospects in this domain are extensive. The utilization of plant extracts for nanoparticle synthesis reduces the reliance on harmful chemicals and facilitates the utilization of agricultural waste or underutilized plant species, thereby establishing a sustainable cycle. The capacity to refine the characteristics of TiO_2_ NPs by choosing plant extracts offers promising prospects for specialized applications in photocatalysis, environmental remediation, antimicrobial therapies, and biomedical devices [40].

The prospects of this research appear favorable. Improvements in comprehending the function of various phytochemicals in nanoparticle formation may result in more predictable and controllable methodologies. Furthermore, integrating plant-mediated synthesis with alternative green technologies, such as solar or microwave-assisted synthesis, may augment efficiency and scalability.

This review focuses on environmentally friendly methods for synthesizing TiO_2_ NPs. It also analyses the vast range of plant species that can be used in this process. Utilizing green methods not only decreases the expense of synthesis but also lessens the necessity for dangerous chemicals and promotes environmentally friendly synthesis. The novelty of this research comes in its in-depth comparison of multiple plant-based green synthesis strategies, stressing the specific impact of distinct plant species on the morphology, size, and functional aspects of TiO_2_ NPs. Unlike previous studies that focused on a limited range of plant extracts or conventional synthesis methods, this review introduces the concept of tailoring nanoparticle properties through the selection of specific plant metabolites, offering insights into how the unique biochemical composition of each plant can optimize TiO_2_ NPs for applications. This strategy presents new paths for increasing nanoparticle efficacy in sectors like photocatalysis and antibacterial activities. These discoveries could motivate researchers to explore more diversified natural resources and develop improved, secure, and cost-effective nanomaterial production processes for a wider range of industrial and biological applications.

This paper specifically examines the distinctive characteristics that come from the output of this environmentally friendly technique, which enhances its suitability for certain applications such as photocatalysis and antimicrobial uses. These remarkable discoveries are likely to inspire researchers and attract new individuals to further investigate and expand the potential applications of natural resources and the development of advanced and more secure techniques for creating nanomaterials. Figure 2 gathers the main approaches applied in the preparation of TiO_2_ nanoparticles, including the green method.

## 2. Synthesis of TiO_2_ NPs from Green Plant Extracts

The synthesis of TiO_2_ NPs presents challenges that can impact their structure, properties, and performance, especially for applications like photocatalysis, solar cells, and sensors. Attaining exact control over phase formation during synthesis is difficult [41,42]. Methods such as sol–gel synthesis, hydrothermal processing, and chemical vapor deposition (CVD) can be intricate and costly, complicating the scalability of manufacturing for commercial applications [43]. Synthesizing TiO_2_ at the nanoscale can markedly augment its features, such as elevated surface area and enhanced photocatalytic activity. Nonetheless, regulating the dimensions and consistency of nanoparticles poses significant challenges [44]. A limited number of pilot-scale studies have demonstrated the feasibility of scaling up such processes [45]. However, significant bottlenecks persist. Moreover, unlike conventional chemical synthesis, green methods often suffer from lower production efficiency and require careful optimization of pH, temperature, and precursor concentration to maintain consistency [46]. Standardization of extract preparation and the establishment of quality control protocols remain critical hurdles for scaling up these methods reliably. Until these challenges are systematically addressed, the commercial viability of green TiO_2_ synthesis remains limited to small-scale applications [47].

Large-scale productivity is further constrained by slower reaction kinetics in comparison to chemical synthesis [48]. Although green synthesis of TiO_2_ is economically appealing and theoretically feasible, practical scale-up is still in early stages. The standardization of plant extracts, the repeatability of the process, clogging, and yield optimization are major obstacles. To transfer lab-scale gains to industry, future research should concentrate on reactor engineering, extract characterization, and techno-economic modelling.

The synthesis of preferred TiO_2_ phases frequently necessitates high-temperature processing, resulting in elevated energy consumption and production expenses [49]. Certain synthesis pathways utilize hazardous solvents or precursors, presenting environmental and safety issues. The pursuit of more environmentally friendly and sustainable synthesis processes remains a persistent task [50]. Plant extracts’ functional groups balance reduction, capping, and stabilization to control the synthesis of TiO_2_ NPs. By giving electrons to Ti^4+^ surface sites, -OH groups function as reducing agents (such as polyphenols and flavonoids) and start nucleation. Smaller particle sizes are preferred due to the large number of tiny nuclei produced by rapid reduction. Adsorbing onto TiO_2_ surfaces, bulky biomolecules such as polysaccharides (found in aloe vera, for example) physically prevent aggregation and restrict development. As a result, NPs have a large surface area and are stable. Surface defects are covered by strong Ti–O bonds made by –OH and –COOH groups (found, for example, in Cannabis sativa), which inhibit oxidative degradation and Ostwald ripening. Green tea polyphenols and other extracts high in -OH groups quickly reduce Ti^4+^, generating a large number of nucleation sites. Growth is limited to sub-10 nm diameters because of competition for scarce Ti^4+^. Atoms can organize into ordered lattices due to slow ion release via –COOH complexation (e.g., aloe vera extracts), which promotes the production of anatase [51]. By forming compounds with a Ti^4+^ center (like citric acid),–COOH groups slow down nucleation through regulated ion release. This promotes consistency by resulting in fewer, well-defined nuclei. Citrus peel is one example of an extract that is rich in –COOH and forms stable Ti–carboxylate complexes that tightly cap NPs and inhibit growth [52]. Certain crystal facets are stabilized by functional groups. For instance, extracts rich in amines encourage rutile under calcination, whereas extracts high in –OH favor anatase [53].

**Figure 3:**
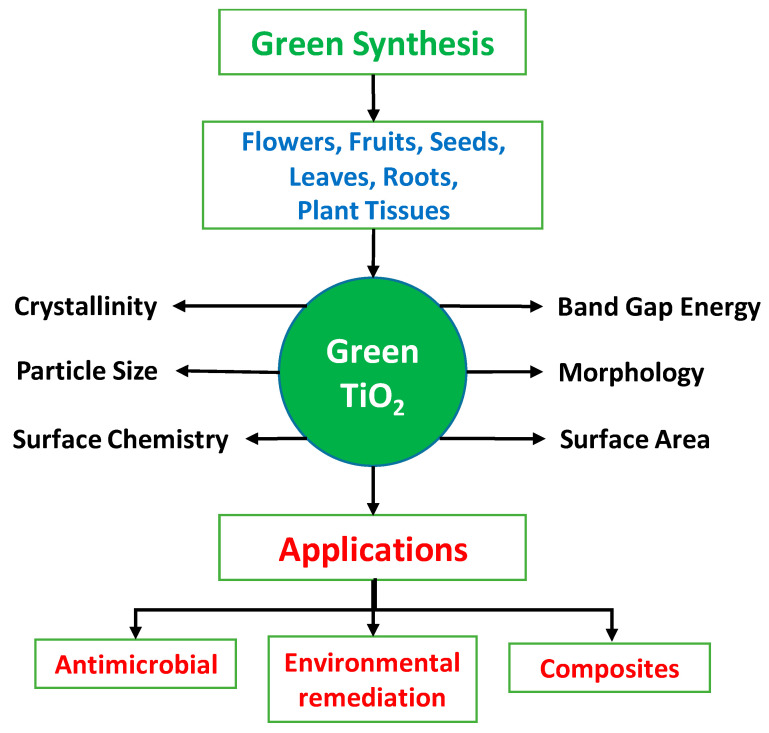
Illustration of TiO_2_ green synthesis and its applications.

The chemical approaches for the synthesis of TiO_2_ NPs have been found to be harmful to the environment, and the synthesis demands high temperatures and pressure, limiting TiO_2_ mass manufacturing. As a result, green nanotechnology has been investigated as an alternative and environmentally responsible strategy for TiO_2_ NPs synthesis since it employs reducing agents obtained from plants sources, and the same reducing agent may be used for the synthesis of numerous metallic compounds. The use of plants, their waste materials, fruit extracts, and microorganisms in synthesis reduces the use of hazardous and costly chemicals [54]. Furthermore, it can be used for low-cost mass synthesis of TiO_2_ NPs [55]. Green synthesis approaches are therefore critical for producing stable, suitably sized, and dispersible NPs with low energy consumption [56]. The advantages of safety and feasibility commonly lead to the use of plant extract as a significant component in the production of TiO_2_ NPs. Plants contain proteins, carbohydrates, enzymes, phenolic acids, and alkaloids, all of which perform reduction and stabilization activities throughout synthesis. Moreover, leaves have a high concentration of metabolites, and they are widely used in green synthesis. The utilization of plant extracts in the processes of nanoparticle production shows more stability due to their higher reaction rate as a reducing agent (capable of reducing metal or metal oxide ions) and as a stabilizing agent, surpassing that of fungus, bacteria, or other organisms. In addition, plant extract gives a significant benefit due to the organism’s need for precise control over cell culture. The green technique is a viable option for scaling up the production of well-dispersed NPs in an industrial setting [24]. Figure 3 shows the sources of extracts for green synthesis of TiO_2_ and its applications.

There are many recent examples of successful synthesis procedures where plant extracts were used, e.g., TiO_2_ NPs synthesized from several *Commenlina benghalensis* plant concentrations [57]. Spherical and agglomerated NPs with a 30–200 nm particle size dispersion and 3.69–3.80 eV band gap were produced. The materials’ surfaces became smoother and more spherical as plant concentration rose. At the greatest concentration, surface area increased from 18 to 174 m^2^/g. The authors of [52] created spherical anatase TiO_2_ NPs with a size range of 15–28 nm utilizing peels, flowers, and leaves. Different extracts affected the size and optical characteristics of the materials. *Tulbhagia violacea* was utilized by Mbenga et al. [58] to create irregular and rectangular NPs. These anatase-structured materials were highly crystalline and varied in shape. Shimi et al. [59] prepared spherical TiO_2_ materials from *Phyllanthus niruri* leaves with a band gap of 2.8 eV. Despite several works of research showing a lower TiO_2_ band gap, there is no specific synthesis pattern to explain it. Perhaps some phytochemicals found in certain plants could affect optical qualities like excitation and band gap shifts. Kaur et al. generated irregularly shaped TiO_2_ NPs with 303 nm particle sizes and 2.1 eV band gap from *Alcea* plant flowers [60]. Aravind et al. prepared TiO_2_ NPs capped and reduced with jasmine flower extract. The rutile phase was confirmed by XRD, while SEM showed randomly organized spherical TiO_2_ NPs having a size of 31–42 nm. UV–Vis measurements revealed the excitation wavelength at 385 nm [61]. Sethy et al. synthesized TiO_2_ NPs from *Syzygium cumini* leaf extract. A strong absorption peak at 356 nm and 3.48 eV band gap were found. Synthesized TiO_2_ NPs were round, irregular, and had an average size of 18 nm in diameter. They removed lead from wastewater with 82.53% efficiency [62]. Arabi et al. formed irregular and spherical TiO_2_ NPs with under 15 nm particle sizes using *Thyme* and *Alcea* [63]. Thakur et al. synthesized spherical, 124 nm sized TiO_2_ NPs from *A. indica* leaf extract; UV–Vis confirmed absorption in the range wavelength of 270–320 nm [64]. *Pomegranate* peel was used to synthesize TiO_2_ NPs, which disinfected water without harming the environment [65].

## 3. The Mechanism of Synthesis of TiO_2_ NPs Prepared with the Use of Green Methods

**Figure 4:**
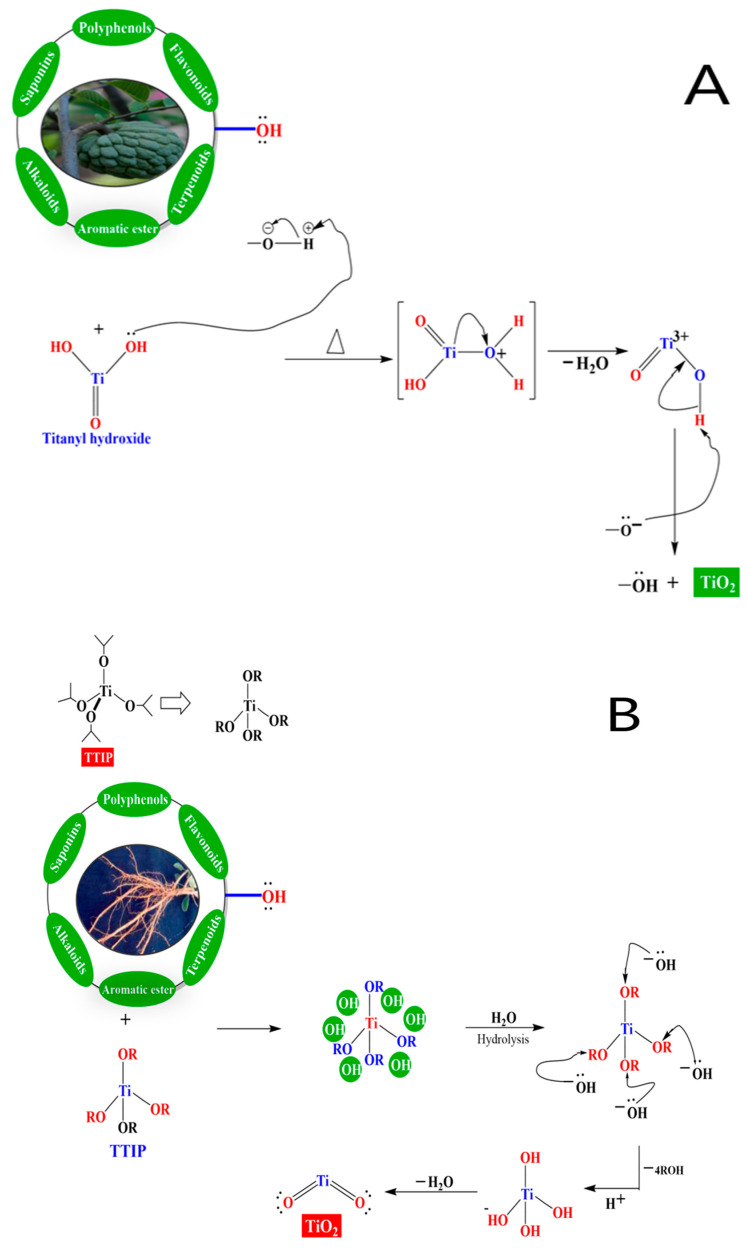

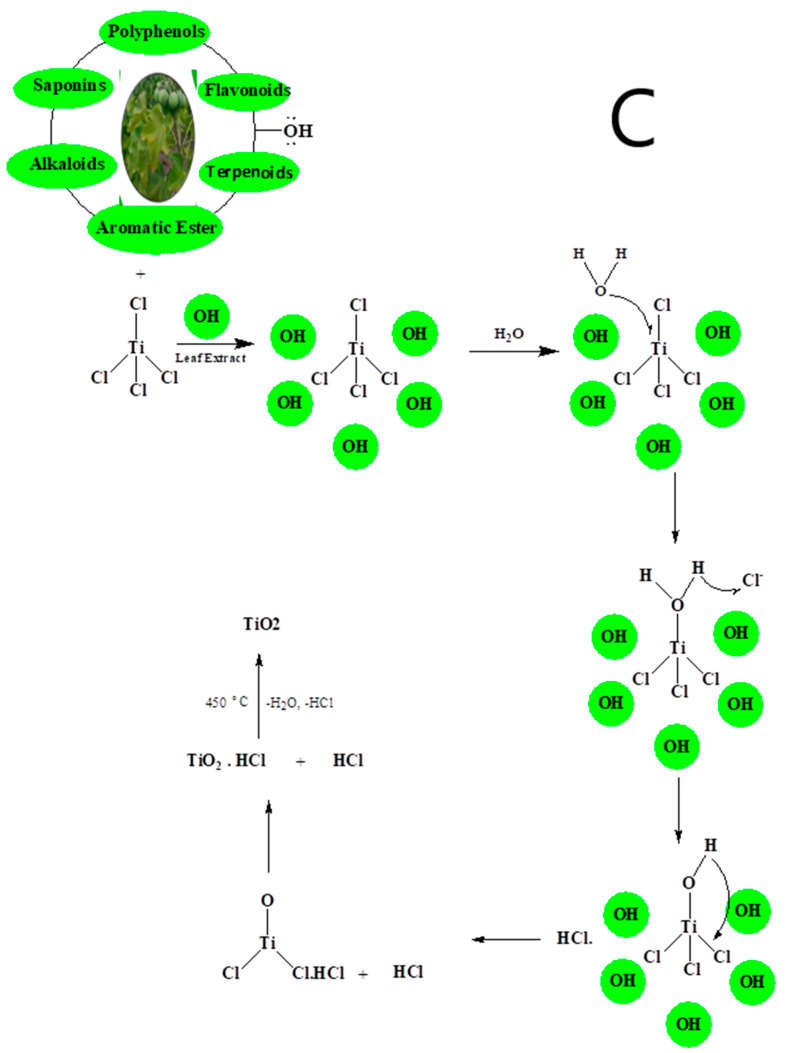
Possible reaction mechanism for the formation of biosynthesized TiO_2_ NPs from different Ti precursors ((**A**): Titanyl hydroxide, (**B**): TTIP, and (**C**): TiCl_4_) [69].

Recently, researchers have focused on understanding the mechanisms involved in green synthesis of TiO_2_. Currently, the precise process by which NPs are produced through plant extract is not fully understood. However, numerous studies have demonstrated that certain biomolecules found in plant materials, such as alkaloids, polysaccharides, alcoholic, and phenolic compounds, may play a role in the creation, stabilization, and reduction of these NPs [66]. The formation of NPs is influenced by the capacity of plants to reduce ions and the reduction potential of these ions. This process is dependent on the presence of biomolecules in plants. Nasrollahzadeh and Sajadi [67] have identified a potential method for the bio-reduction of titanyl hydroxide, as depicted in Figure 4.

Initially, they conducted FTIR analysis to ascertain the presence of biomolecules. Consequently, the researchers determined that the hydroxyl groups of phenolics in the root extract of *E. heteradena Jaub* are accountable for the reduction of TiO(OH)_2_ and serve as capping ligands on the surfaces of the NPs. Moreover, the FT-IR data clearly indicates distinct variations in the position and morphology of signals, suggesting a connection between the phytochemicals and the sites involved in the green synthesis process. This implies that polyphenolics are likely to be adsorbed onto the surface of the NPs. Biomolecules such as flavonoids, alkaloids, polyphenols, terpenoids, heterocyclic compounds, and polysaccharides have been found to play important roles in reducing, stabilizing, and acting as capping agents for highly uniform NPs. The authors of [68] demonstrated that carboxylic acids found in *Aloe vera* leaf extract, which are identified by broad -OH stretching in FTIR analysis, indicate the presence of terpenoids, flavonoids, and proteins. These compounds can aid in the formation and production of biosynthesized TiO_2_ NPs. Additionally, they act as both a capping and reducing agent during the biosynthesis process. The work conducted by Goutam et al. [69] demonstrated the existence of hydroxyl groups in *Jatropha curcas* L., suggesting that the surface of TiO_2_ NPs synthesized through biological means was covered by phenols or polyphenolic tannins. Even though the biochemical mechanism is unknown, those biochemical substances are crucial to the reduction, stabilization, and capping of the reaction. Understanding the mechanisms will boost nanoparticle productivity. Figure 4 presents the formation mechanism of TiO_2_ NPs obtained from two types of precursors: titanyl hydroxide and titanium tetrachloride, respectively.

## 4. Limitations of Green Method for NPs Synthesis

The process of green synthesis involves the utilization of environmentally friendly and biocompatible ingredients as both reducing agents and stabilizing agents during the manufacture of NPs. However, there are several limitations to green synthesis, some of which are the following:

1. Green synthesis is hindered by the inability to precisely manipulate nanoparticle (NP) size and form depending on reaction duration, temperature, pH, and reactant concentration [70].
2. Green synthesis has a lower NPs yield than conventional methods. The instability of the reducing agent, poor precursor ion reduction, and agglomerates can cause low yields. When industrial nanoparticle demand is high, insufficient production output can be a major issue [71,72].
3. Green synthesis can struggle to scale up production to synthesize large volumes of NPs. Scalability may be constrained by the limited availability and unpredictability of natural extracts or microbes used as reducing agents [24,71].
4. Green synthesis also faces nanoparticle stability and surface chemistry issues. Nanoparticle surface chemistry greatly affects their interactions with biological systems and other materials. The use of natural extracts or microorganisms as reducing agents can make nanoparticle surface chemistry difficult to manage. Natural extracts used as reducing agents can also absorb contaminants onto nanoparticle surfaces, changing their characteristics [73].

## 5. Characterization of TiO_2_ NPs

The primary objective of nanoparticle characterization is the examination of critical parameters including charge, size, shape, and degree of aggregation. Various techniques are frequently employed to characterize NPs, including Fourier transform infrared spectroscopy (FTIR), X-ray photoelectron spectroscopy (XPS), energy-dispersive X-ray spectroscopy (EDS), and scanning electron microscopy (SEM). Additionally, transmission electron microscopy (TEM) and dynamic light scattering (DLS) are also utilized. 

## 6. Optical Properties of TiO_2_ NPs

Quantification of the amount of light that is absorbed and scattered by a sample, e.g., TiO_2_, is a technique that is frequently utilized these days. When a sample is positioned between the UV–Vis light source and the detector, light is permitted to fall on and go through the sample. The intensity of the beam of light is measured both before and after it passes through the sample [74]. UV–Vis spectroscopy can be considered as a method that not only confirms the presence of TiO_2_ NPs in aqueous solution as an effect of green preparation but first shows the absorption intensity (and range) of the resulting material. Different scientific research indicated that titanium ions are reduced, and TiO_2_ NPs are generated within a few hours at room temperature. The solution turns light green when titanium ions decrease. Absorption peaks at 245–265 nm are seen in plant extract solutions used for TiO_2_ NPs preparation, while chemically synthesized TiO_2_ NPs exhibit a little absorbance shift, with a peak at 303 nm (λ-max).

## 7. Functional Group Characterization of TiO_2_ NPs Prepared Using Green Method

Fourier transform infrared spectroscopy (FTIR) is employed to analyze the impact of reducing and stabilizing agents on the surface chemistry of nanomaterials. Additionally, it plays a crucial role in elucidating the chemical bonding present within materials and the functional molecules that are attached to the surface of nanomaterials. In FTIR analysis, the samples undergo exposure to infrared (IR) radiation. The IR radiation subsequently exerts influence on the atomic vibrations of a molecule within the specimen, leading to the selective absorption and/or transmission of energy [75].

FT-IR spectroscopy was utilized to analyze the synthesized TiO_2_ NPs obtained using various methods, including green synthesis. The purpose was to identify the potential functional groups contained in the synthesized samples. The FT-IR spectra were recorded within the wavelength range of 4000 to 400 cm^−1^. It has been observed that titanium dioxide NPs synthesized using a green technique exhibit a comparable FTIR spectrum. Typically, samples exhibiting a green color demonstrated an absorption band within the spectral region of 650–700 cm^−1^, indicative of the stretching link between titanium (Ti) and oxygen (O) atoms (Ti-O-Ti). The absorption peak observed within the wavenumber range of 650–700 cm^−1^ is recognized as the distinctive peak of the anatase phase of TiO_2_ NPs, as reported in the literature [62,76]. The observed peaks within spectral range of 1000–1300 cm^−1^ can be attributed mostly to Ti-O-Ti vibrations, indicating the presence of O-Ti-O bonding. Additional carbon functional groups, such as CH, CH_2_, CH_3_, or O-CH_3_, may also be observed within the mentioned frequency range. These functional groups could potentially originate from plant extracts. An intriguing distinction may be observed in the synthesis processes, namely in the choice of precursors employed during the synthesis process. The distinctive characteristic of green TiO_2_ NPs can be attributed to the hydroxyl (-OH) groups found in leaf extracts of plants, which are present because of the prevalence of phenols. The likely explanation for the presence of the phenolic group in both extracts is the presence of polyphenolic tannins. These tannins are found on the surface of TiO_2_ NPs that were synthesized using environmentally friendly materials [77,78,79]. Surface-bound biomolecules derived from plant extracts improve the long-term stability of TiO_2_ NPs by inhibiting aggregation through steric and electrostatic interactions, as well as chelation [20]. They enhance biocompatibility by decreasing reactive oxygen species (ROS) and cytotoxicity by surface passivation, as shown by PEG-coated TiO_2_ nanoparticles [80]. These biomolecules might improve visible-light catalytic activity by dye sensitization (e.g., chlorophyll) or electron transfer (–OH groups); nevertheless, excessive organic layers can block active sites. Functional groups confirmed by FTIR (e.g., C=O, –OH stretching) are associated with these effects, as seen in TiO_2_ synthesized from orange peel extract [20].

**Figure 5:**
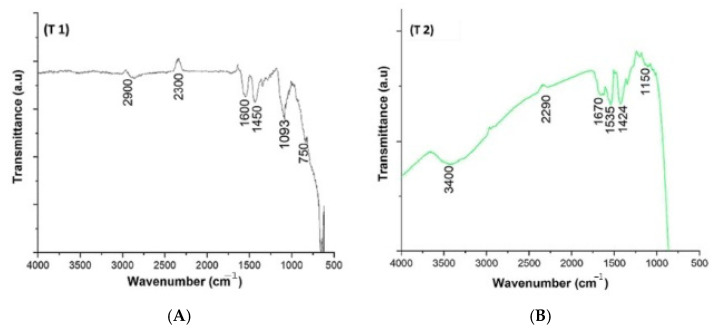

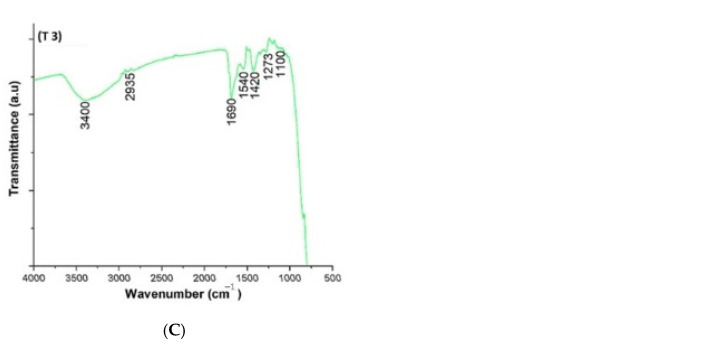
FT-IR absorption spectra of chemically synthesized TiO_2_-NPs (**A**)—(T1), (**B**)—(T2), and (**C**)—(T3) are synthesized by the plant extracts of *T. portulacastrum* and *C. quinoa*, respectively. Reprinted with permission from ref. [76] with permission of Elsevier.

Figure 5 shows an example of the FT-IR spectra of TiO_2_ NPs synthetized with the use of plant extracts.

## 8. Morphology of TiO_2_ NPs

The scanning electron microscope (SEM) is an instrument that utilizes a focused electron beam to scan across a surface to generate a picture. The scanning electron microscope (SEM) and transmission electron microscope (TEM) vary fundamentally in their operational principles. While TEM involves the transmission of electrons through a sample, SEM operates by directing a beam of electrons onto the surface of the sample and collects secondary electrons (SE) and/or backscattered electrons (BSE) in detectors to create an image. Through interactions between the electron beam and the sample, an image is generated pixel by pixel. The SEM preserves the three-dimensional topography of the material because of its greater depth and width compared to the TEM [81]. The green TiO_2_ NPs are composed of porous structures that exhibit relatively large crystals, displaying an intriguing and distinctive surface appearance. Due to their porous architecture, TiO_2_ NPs exhibit significant potential for revolutionary applications within the environmental applications. The distinctive composition of the leaf extracts of the plant material is attributed to the presence of phytochemicals, including terpenoids, steroids, polyphenols, flavonoids, alkaloids, antioxidants, free amino acids, and tannins that affect the topography and morphology of TiO_2_ NPs. In this context, the investigation of NPs morphology with the use of microscopy techniques is indispensable. In a recent study conducted by Shimpi et al., the authors successfully synthesized TiO_2_ NPs using a solution of titanium tetra-isopropoxide (TTIP) as a precursor, together with a leaf extract obtained from *Murraya koenigii*, often known as the Curry tree. The researcher utilized an aqueous extract of a plant and an ethanolic leaf extract to synthesize TiO_2_ NPs. A comparison was made between the obtained results and the standard parameters. It was observed through FESEM and TEM analysis that the NPs produced from the ethanolic leaf extract exhibited a perfectly spherical shape with a smaller size range of 2–15 nm. In contrast, the NPs synthesized using the aqueous leaf extract displayed a scattered spherical crystallite shape with a larger size range of 15–30 nm. In addition, the author also conducted a study of TiO_2_ NPs synthesized using chemical and green processes, revealing a disparity in their respective band gap energies of 4.3 eV and 4.5 eV [82].

**Figure 6:**
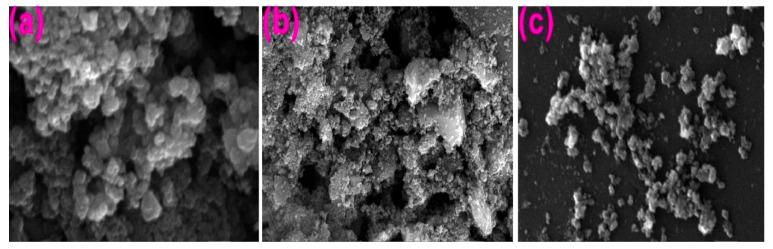
SEM and EDX spectrum of (**a**) *Ceaspina pulcherrima* flower, (**b**) *Nervila aragona* leaf, and (**c**) *Manihot esculante* peel extract TiO_2_ NPs. Reprinted from ref. [52].

The chemical production process exhibits the highest percentage of yield, whereas the percentage of TiO_2_ NPs is comparatively lower. This method of synthesis can be considered environmentally friendly and sustainable. To obtain TiO_2_ NPs from environmentally friendly sources, a method that eliminates precursors or reduces synthetic chemicals is needed [62,76,83]. Figure 6 presents the morphology (SEM) and chemical composition (EDX) of TiO_2_ NPs obtained using flower, leaf, and peel extracts of various plants.

## 9. Analysis of Crystallographic and Phase Structure of TiO_2_ NPs

The technique employed for the characterization of NPs is widely utilized. X-ray diffraction (XRD) is a commonly used technique in materials science that offers valuable insights on the structural characteristics, dimensions, composition, and lattice parameters of a material. The powdered material, which has been thoroughly dried, is utilized in X-ray diffraction (XRD) analysis.

The TiO_2_ NP standards describe the tetragonal anatase phase of crystallite, with space group I41/amd and lattice parameters a = b = 3.792 Å and c = 9.554 Å. The TiO_2_ NPs demonstrate a prominent peak observed at the 2θ value ranging from 24° to 28°, with a specific occurrence at 25.6°, which corresponds to the anatase (101) crystallographic plane. The XRD graphs revealed additional minor peaks at 2θ values of (38°, 48°, 54°, and 63°), corresponding to the (112), (200), (211), and (204) planar angles of the anatase phase. The XRD pattern of crystals indicates that this value is associated with the anatase form of TiO_2_ NPs, along with the phases of brookite and rutile. Furthermore, it is worth noting that the observation of the anatase form of TiO_2_ NPs at a certain plane angle is consistent across the study community. The property of TiO_2_ NPs, namely the 2θ peak at 25.6°, is not influenced by the synthesis process, as demonstrated in a study conducted by [76]. The X-ray diffraction (XRD) pattern of TiO_2_ NPs synthesized using plant extracts from *T. portulaca* strum *and C. quinoa* was compared with the sol–gel approach. The results demonstrate that the synthesis method does not influence the crystallinity of TiO_2_ NPs. In addition, [83,84] synthesized TiO_2_ NPs using extracts from *Carica papaya* and *Cymbopogon citrates*, respectively, and the XRD study yielded similar results. In addition to crystallinity, the average particle size of green TiO_2_ NPs has mostly been determined using the Debye–Scherrer equation (1) after successful synthesis [69,85]. The Scherrer equation is commonly regarded as the primary and extensively employed equation for determining particle size through the utilization of 2θ and FWHM values derived from X-ray diffraction (XRD) data.

D = Kλ βcosθ(1)

**Figure 7:**
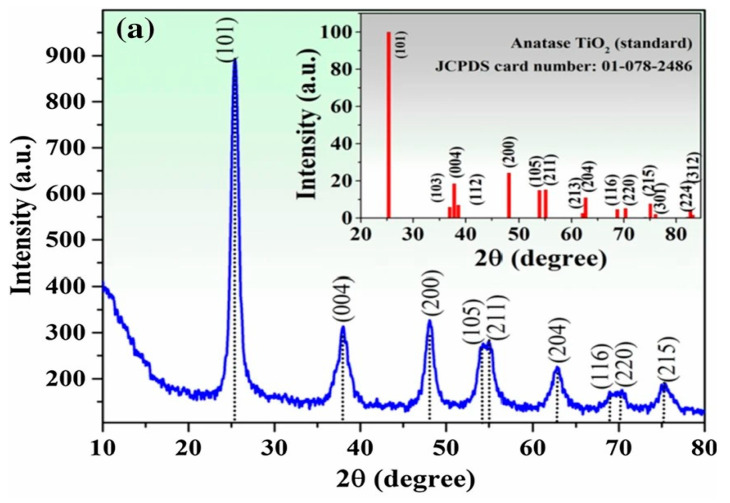
XRD pattern of green synthesized TiO_2_ NPs with JCPDS card. Reprinted with permission from [82], with permission of IOP publisher.

**Figure 8:**
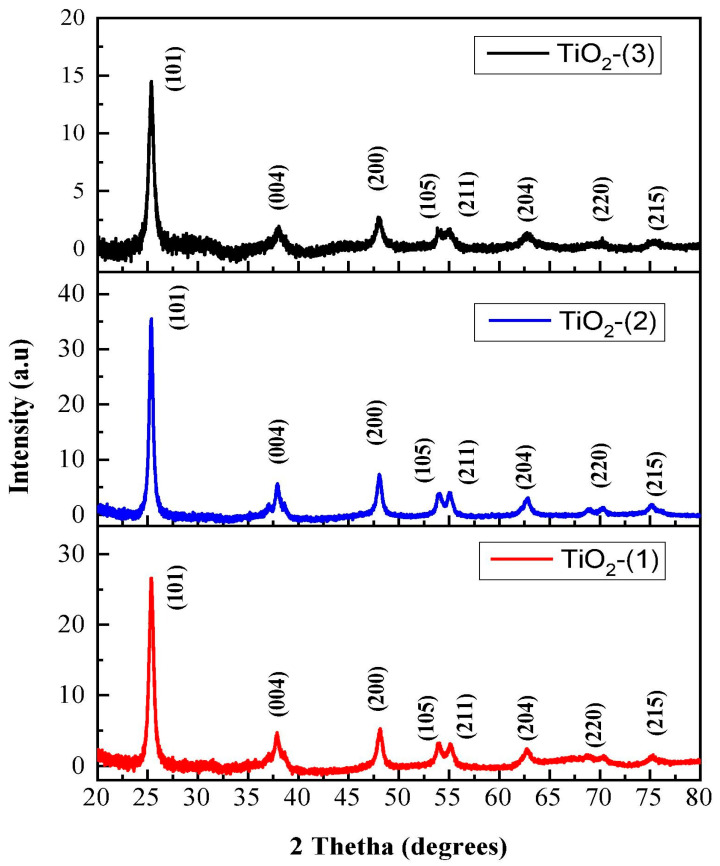
XRD of TiO_2_(1), TiO_2_(2), and TiO_2_(3) at different *T. violacea* bulb extract volumes of 10, 20, and 40 mL, respectively. Reprinted with permission from ref. [58] with permission of Elsevier.

In the given equation, the symbol D is used to indicate the particle size, specifically the diameter. The symbol K represents Scherrer’s constant, which has a value of 0.9. The symbol λ denotes the wavelength of the X-ray source, specifically 0.15406 nm. The symbol b is used to represent the full width at half-maximum intensity (FWHM) in radians. Lastly, the symbol q is utilized to designate the peak locations, measured in radians. Figure 7 presents the XRD patterns of green synthesized TiO_2_ NPs with a JCPDS card and Figure 8 presents the XRD patterns of TiO_2_(1), TiO_2_(2), and TiO_2_(3) at different *T. violacea* bulb extract volumes of 10, 20, and 40 mL, respectively.

## 10. Applications for Green TiO_2_ NPs

TiO_2_ NPs synthesized using green methods have been investigated for their potential applications in dye-sensitized solar cells (DSSCs), as well as in various biomedical fields such as drug delivery, imaging, and antimicrobial treatments. Additionally, these nanoparticles have shown effectiveness in removing heavy metals and other pollutants from polluted water and soil. Their photocatalytic characteristics allow for the decomposition of organic pollutants, while their large surface area facilitates the adsorption of metal ions. This distinctive behavior of TiO_2_ NPs differentiates it from other metallic nanoparticles. The comparison of the application of green synthesized TiO_2_ NPs and other green synthesized metal and metal oxide nanomaterials is presented in Table 2.

One of the important current applications of TiO_2_ NPs is the ability to produce reactive oxygen species (ROS) that subsequently take part in water purification processes. ROS generated by titanium dioxide can effectively degrade organic pollutants and disinfect water, making it a promising technology for clean drinking water production. Additionally, the future applications of titanium dioxide’s ROS include its potential use in cancer treatment, as ROS have been shown to induce cell death in cancer cells while sparing healthy cells.

## 11. Antimicrobial Activity of Green Synthesis TiO_2_ NPs

**Figure 9:**
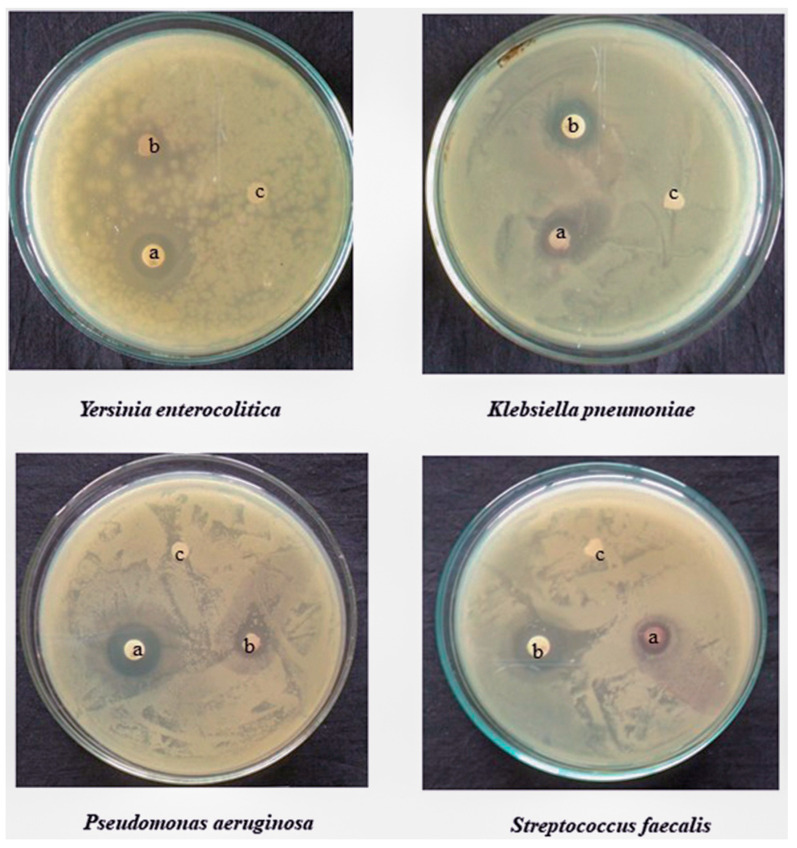
Antimicrobial activity of *Trigonella foenum-graecum* derived TiO_2_ NPs against different microorganisms depicting zones of inhibition of (a) positive control, (b) TF-TiO_2_ NPs, and (c) dimethyl sulfoxide control. Reprinted with permission from ref. [56], with permission of Elsevier.

Antimicrobial drugs inhibit or eradicate the proliferation of germs. Extensive research has been conducted on the antibacterial characteristics of metal oxide NPs, specifically TiO_2_, yielding encouraging outcomes. *Psidium guajava*-derived TiO_2_ NPs were evaluated for their antimicrobial activity against human pathogenic bacteria using the disc diffusion method. The antioxidant activity of TiO_2_ NPs is significantly higher than that of ascorbic acid. *Staphylococcus aureus* (*S. aureus*) exhibited the highest level of zone inhibition, measuring 25 mm, while *E. coli* had a little lower zone inhibition of 23 mm. The synthesized TiO_2_ NPs exhibited superior antibacterial efficacy compared to the typical antibiotic disc, tetracycline, resulting in a considerable reduction in the effectiveness of antibiotics in protecting against bacterial infections [86]. The antibacterial effectiveness of TiO_2_ NPs synthesized using *Azadirachta indica* leaf extract was investigated against various pathogens, including *E. coli*, *Klebsiella pneumonia*, *Salmonella typhi* (*S. typhi*), and *Bacillus subtilis* (*B. subtilis*). The NPs exhibited the lowest Minimum Inhibitory Concentrations (MIC) value of 10.42 μg/mL against *E. coli* and *S. typhi*. However, the lowest Minimum bactericidal concentration (MBC) value of 83.3 μg/mL was seen against *K. pneumonia* [64]. The synthesis of green TiO_2_ NPs from *Mentha arvensis* leaf extract results in the manifestation of antifungal and antibacterial properties against several microorganisms including *Aspergillus niger* (*A. niger*), *Proteus vulgaris* (*P. vulgaris*), *E. coli*, *Arthrographis cuboid*, *S. aureus*, and *Aspergillus fumigatus* (*A. fumigatus*). The TiO_2_ NPs exhibit remarkable antibacterial efficacy against *P. vulgaris*, owing to their ability to dissolve the outermost bacterial membrane, which is the primary cause of bacterial demise [87]. Various laboratory techniques were employed to evaluate and test the antibacterial properties of an extract derived from a valuable chemical. In addition to commonly used and essential techniques like agar dilution and disc diffusion, other methods such as poisoned food are employed for antifungal testing. The antibacterial activity of green synthesized TiO_2_ NPs is attributed to the biomolecules derived from the *Kniphofia foliosa* root extract. Furthermore, it donates an extra electron to the surface of TiO_2_, resulting in the formation of superoxide radicals O_2_^•−^. This, in turn, leads to the production of reactive oxygen species (ROS) within the bacterial cell. This ROS disrupts the integrity of bacterial cell membranes. Elevating the production of the superoxide radical also enhances the generation of reactive oxygen species (ROS) within the bacterial cell. The generation of electrons from TiO_2_ NPs is responsible for the antibacterial action exhibited against both Gram-negative and Gram-positive bacteria strains [88]. The significant porosity and average particle size of TiO_2_ NPs further promote bacterial mortality. Bulk materials lack the ability to connect with cellular components such as proteins, carbohydrates, nucleic acids, and fatty acids. However, small NPs can interact effectively due to their large specific surface area (SSA). Consequently, they exhibit higher toxicity and induce detrimental effects on bacterial pathogens. Moreover, due to its minuscule dimensions, it can penetrate cells and inflict damage upon them. Particle size can have an impact on the rate at which a substance dissolves. The little particle dissolves more rapidly due to its extensive surface area. The pharmaceutical industry utilizes this understanding when formulating new drugs, as substances with a larger surface area (smaller grain size) may undergo digestion at a faster rate. Due to the production of free radical oxides and peroxide, TiO_2_ NPs have potent antibacterial activity and broad reactivity against various microbial pathogens. Consequently, they have been shown to have a beneficial antimicrobial effect. The orange peel extract facilitates the eco-friendly production of TiO_2_ NPs. These NPs exhibit a superior inhibitory effect on the growth of *P. aeruginosa* compared to *E. coli* and *S. aureus*. Furthermore, they demonstrate an enhanced ability to combat cancer when tested against the A549 lung cancer cell line. The presence of TiO_2_ NPs in quantities ranging from 40 to 400 mg can induce cell membrane damage, oxidative stress, decreased levels of glutathione, and elevated levels of lipid peroxidation stress [89]. Figure 9 presents antimicrobial activity of *Trigonella foenum-graecum* TiO_2_ NPs against different microorganisms. Some examples of NPs synthesis, characterization, and application by using different plant extract are shown in Table 3.

## 12. Mechanism of Antimicrobial Activity

Although recent papers have postulated and explained various routes of cell death using reactive radicals, the precise mechanism by which NPs exhibit their antibacterial effects remains unknown and requires additional research. Generalizing the precise method by which NPs kill germs is challenging because to the variability in the types of NPs, strains, and environmental conditions employed in antibacterial experiments. Various NPs display varying antibacterial activity against different strains. Therefore, it is crucial to include these subjects in the debate forum to conduct a thorough investigation and clarification of the precise mechanism of action of NPs. Several difficulties, such as the entry of NPs into cells with varying structural integrity, need to be clarified. Furthermore, the precise mechanism by which NPs affect protein breakdown, DNA damage, and the modification of fundamental metabolic processes in cells remains largely unclear.

The main mechanisms by which NPs exhibit antibacterial activity, as suggested in the literature, are as follows: (a) the induction of oxidative stress through the production of ROS [93]—this oxidative process leads to lipid membrane peroxidation, resulting in damage to proteins and DNA within bacterial cells; (b) the release of metal ions from metal or metal oxide NPs, which can penetrate bacterial cell walls and directly interact with nucleic acids and proteins containing –SH, –NH, and –COOH groups, ultimately leading to cell death [94,95].

**Figure 10:**
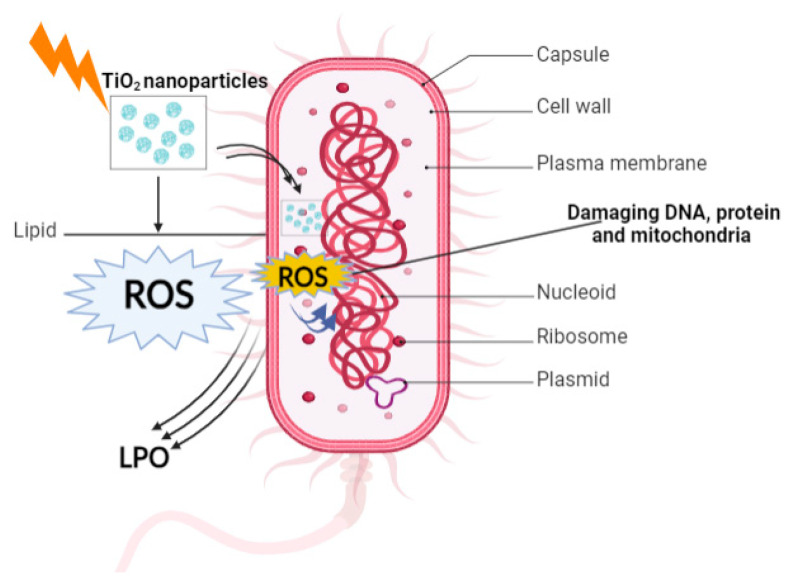
Antibacterial action mechanism of TiO_2_ NPs.

In 1985, ref. [96] published the first results on the antibacterial and photoelectrochemical properties of TiO_2_ powders loaded with platinum (TiO_2_/Pt). These powders were found to effectively kill Lactobacillus acidophilus, Saccharomyces cerevisiae, and *Escherichia coli* (*E. coli*) bacteria. Researchers have made significant efforts to enhance the photocatalytic bactericidal activity of TiO_2_ NPs. TiO_2_ nanostructures possess a broad range of uses in various industries, environmental processes, and energy-related applications. These include but are not limited to water purification, food preservation, the breakdown of dyes, chemical sensing, dye-sensitized solar cells, and the creation of antimicrobial agents. TiO_2_ doped with metals and non-metals that respond to visible light have been found to have bactericidal effects on various bacterial species, such as Gram-negative *E. coli*, *Acinetobacter baumannii*, and *Shigella flexneri*, as well as Gram-positive *S. aureus*, *Bacillus subtilis*, *Listeria monocytogenes*, and *Bacillus anthracis spores* [97]. These photocatalysts can sterilize dangerous germs, therefore effectively stopping the transmission of diseases caused by microbes. A general scheme showing the antibacterial action mechanism of TiO_2_ NPs is presented in Figure 10.

## 13. Green TiO_2_ as a Photocatalyst for the Degradation of Dyes

Green TiO_2_ has emerged as a promising photocatalyst for the degradation of dyes due to its enhanced efficiency and environmental friendliness. Its unique composition and structure absorb light efficiently, generating reactive oxygen species that break down dye molecules. Green TiO_2_ reduces harmful by-products, making it a sustainable textile wastewater treatment solution. The photocatalytic process of dye molecules by an anatase TiO_2_ catalyst generates highly reactive species like hydroxyl radicals (^•^OH), holes (h^+^), superoxide (O_2_^•−^), and per hydroxy radicals. These species are formed in the solution when the catalyst absorbs radiation with a wavelength of 388 nm. These radicals react with chromophores and auxochromes to mineralize the dye molecule into carbon dioxide, water, and metal complexes [98]. The combined effects of surface area optimization, band structure modification, and crystallinity control, mediated by plant-derived biomolecules, give green-synthesized TiO_2_ nanoparticles (NPs) superior photocatalytic activity when compared to their chemically synthesized counterparts. Because of the chelation of the Ti^4+^ center, plant extracts high in polyphenols (–OH groups) or carboxylic acids (–COOH), like Cannabis sativa (bhang), promote anatase production, slowing hydrolysis and facilitating orderly crystallization. The metastable structure of anatase (band gap ~3.2 eV) improves photocatalytic activity and charge separation. High-temperature calcination (>500 °C) or extracts containing amines encourage rutile (band gap ~3.0 eV), which absorbs more visible light. In contrast to chemically synthesized anatase, jasmine flower extract produced rutile-phase TiO_2_ NPs with a 92% methylene blue degradation efficiency [61]. A high surface area-to-volume ratio and more reactive sites for dye adsorption and degradation (98.2% methylene blue elimination) were provided by the 12.5 ± 1.5 nm particle size of the TiO_2_ NPs made from Cannabis sativa [99]. In microwave-assisted synthesis, L-ascorbic acid produced oxygen vacancies (V_o_) and Ti^3+^ defects, which reduced the band gap and extended light absorption into the visible and near-infrared regions. Under visible light, this faulty TiO_2_ produced 162 μmol g^−1^ h^−1^ of hydrogen [100]. Moreover, the decomposition of –OH, –COOH surface groups, or adsorbed bulky biomolecules upon illumination may be the source of decomposition products that either enhance photoactivity by highly active radicals or hinder photoactivity by free radical scavenging properties in further photocatalytic reactions. Another factor enhancing the degradation on green TiO_2_ may be better adsorption of dyes on the surface of photocatalyst. Finally, the adsorbed molecules may behave as photosensibilization agents, improving the photocatalytic process.

A flower extract produced TiO_2_ NPs with the highest absorbance and lowest band gap of 2.89 eV, according to Rathi [52]. Floral extract-generated TiO_2_ NPs likewise exhibited a maximum degradation of 86% against MB dye. Hiremath et al. [101] produced *Taramindus* TiO_2_ NPs with a band gap of 3.2 eV and a high surface area of 75 m^2^/g. The anatase material underwent testing to assess its ability to degrade Titan Yellow (TY) dye, with a focus on examining different variables. A maximum degradation of 95% was achieved under neutral conditions (pH = 7) with a dye concentration of 10 ppm and 75 mg of TiO_2_ NPs in less than 2 h. Pavithra et al. [102] synthesized TiO_2_ NPs using *Colotropis gigantea* for the purpose of degrading MB dye. A reduction of 96% was observed after one hour of utilizing *Acorus calamus* extract to create spherical and interconnected clusters of TiO_2_ with a particle size of 39 nm, achieving 96% degradation of RhB from its initial concentration [103]. The degradation of Congo Red (CR) dye and MB was also examined using *Myristica fragrans* TiO_2_ NPs. The anatase structured material, which exhibited a blue shift, achieved a maximum degradation of 99% and 97% against CR and MB, respectively. The optimal efficiency was achieved due to the formation of O_2_^•−^ and holes. The authors of [104] investigated the impact of pH, time, dosage, and concentration on the degradation of MB utilizing *Monsonia burkeana* TiO_2_ NPs. They also examined the potential mechanism of degradation. The maximum degradation of 85% for MB occurred at a pH of 10 after 120 min, using a dosage of 60 mg TiO_2_ NPs and a concentration of MB of 20 ppm. However, when testing the material’s capacity to be utilized again, it was found that the TiO_2_ NPs only lasted for one cycle. This suggests that either the catalyst was lost, or the active sites were blocked, leading to a significant decrease in the material’s ability to degrade.

## 14. Mechanism of Photocatalysis

Instead of changing the photocatalyst, light energy is converted into chemical energy to synthesize or break down molecules. When light is applied, electron–hole pairs are created, which starts photocatalytic activity. Holes in the VB will appear when a semiconductor photocatalyst absorbs photons equal to or higher than its E_g_ while the electrons in the VB will be stimulated to the CB. On the other hand, photogenerated holes and electrons are captured by defect sites within the catalyst’s surface and/or in bulk, where they recombine to produce heat or photons [105]. The rate of photogenerated carrier recombination determines the carrier lifespan; rapid recombination results in short lifetimes, which in turn restricts the catalyst’s ability to convert photochemical energy into a useful form [106].

The transfer of electronic charges to the semiconductor catalyst’s outer surface helps to promote interfacial substrates and chemical reactions. When photogenerated electrons in CB interact with the dissolved oxygen at the catalytic surface, they produce superoxide radicals (O_2_^•−^), which then react with the holes on the VB to produce singlet oxygen (^1^O_2_). Photogenerated holes in the VB are effective oxidizers (+1.0 to +3.5 V vs. NHE) and interact with surface moisture (H_2_O or OH^–^) to create H_2_O_2_ and hydroxyl radicals. Reaction between hydroxyl radicals (HO^•^) and hydrogen peroxide (H_2_O_2_) produces protonated superoxide radicals (HOO^•^) [107,108].


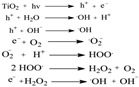


Electrons and holes may recombine within the semiconductor rather than transfer to the catalytic outer surface. One disadvantage of this light-driven method is that recombination of photogenerated electrons and holes might lose most of the energy as heat. In this regard, a greater gap between electrons and holes is required to produce a quick shift in photocatalytic activity and redox chemical processes in the semiconductor.

**Figure 11:**
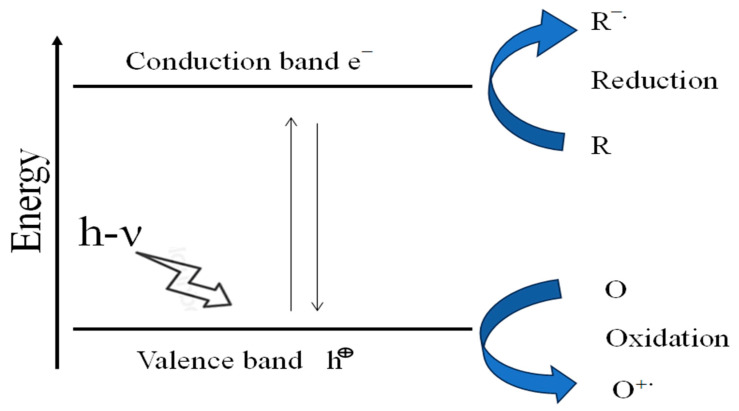
Simplified diagram illustrating the fundamental principles of semiconductor photocatalysis.

Adjusting the intrinsic band structure can help with pollutant adsorption efficiency, photocatalytic efficiency, redox potential, and charge carrier recombination/separation. The key to understanding the photocatalytic process and increasing photocatalytic activity is having a solid understanding of electronic transition [109]. Figure 11 illustrates the fundamental principles of semiconductor photocatalysis.

## 15. Conclusions

This publication presents a review of methods for the synthesis of titanium dioxide nanoparticles using various plant extracts. Numerous possibilities for the synthesis of this nanomaterial are presented, paying special attention to the limitations. Physicochemical properties of nanomaterials obtained by green methods are characterized in terms of their morphology, optical properties, crystallographic structure, and the presence of functional groups. Finally, directions for the application of TiO_2_ nanomaterials are presented, especially as antimicrobial nanomaterials and as photocatalysts. The numerous significant benefits that are associated with these methods are the primary reason for the tremendous support that is being shown for green synthesis. In a wide variety of applications, green synthesis helps to reduce the negative effects that the generated nanoparticles have. In addition to this, it is safe, economically viable, efficient, and environmentally friendly, and it can be easily improved. Due to the reduced utilization of precursors during the green synthesis process, the green technique exhibits a higher level of efficiency in comparison with chemically synthesized nanoparticles techniques. For example, *Psidium guajava*-derived TiO_2_ NPs demonstrated the maximum zone of inhibition at 25 mm against *Staphylococcus aureus*. TiO_2_ NPs obtained with the use of *Calotropis gigantea* showed a reduction of 96% of MB dye after one hour. Because fewer plants are currently being utilized for the environmentally friendly synthesis of TiO_2_ NPs, there is still a great deal of work to be done in this field. On the other hand, these nanoparticles from green synthesis can be used in biomedical applications with low risks, and they are just as compatible with chemically synthesized nanoparticles in other potential applications. TiO_2_ NPs have the potential to be an effective treatment for polluted wastewater as well as soils that have been contaminated with metals. However, the efficacy of the treatment is contingent upon several factors, including the type of metal, the duration of exposure, the dosage, and the form of the nanoparticles. Green synthesis can decrease material expenses by roughly 30–50%. The removal of dangerous substances such as titanium tetrachloride might result in a reduction in raw material expenses by as much as 40%. TiO_2_ NPs synthesized via green methods might exhibit up to 25% more photocatalytic efficiency and 30% enhanced antibacterial activity relative to those produced through chemical synthesis. It is necessary to conduct additional research to improve one’s understanding of the production and utilization of green TiO_2_ NPs.

## Figures and Tables

**Table 1:** Circular economy aspect of chemical synthesis versus specific green synthesis [32,33,34,35].

Features	Traditional Chemical Synthesis	Green-Synthesized TiO_2_ NPs
Material Cost Reduction	Baseline	30–50% lower
Photocatalytic Efficiency	Low	25% higher
Antimicrobial Activity	Low	30% improved
Reusability	Typically, single use	Up to 10 cycles
Waste Utilization	Significant hazardous waste	Reduces plants waste by 90%
Scalability	Easily scalable but less sustainable	Potential for 1 ton/month
Economic Value from Waste	No value recovery from waste	USD 1000 per ton of plants by-products

**Table 2:** Comparison between green synthesized titania nanomaterials and with other green synthesized metal nanomaterials.

Property	*TiO_2_* *NPs*	*Silver* *NPs*	*Zinc Oxide* *NPs*	*Gold* *NPs*	*Iron Oxide* *NPs*
Plant Extracts	Aloe vera, neem, green tea	Cinnamon, neem, green tea	Hibiscus, aloe vera, turmeric	Mangosteen, green tea,	Green tea, eucalyptus, moringa
Photocatalytic Activity	High (under UV/visible light)	Moderate	High (UV light)	Low	Moderate (good under visible light)
Antimicrobial Properties	Moderate	High	Moderate	High	High
Cost of Synthesis	Low	Moderate	Low	High	Low
Effectiveness in Wastewater Treatment	High (removes dyes, heavy metals, organic pollutants)	High (antimicrobial, removes organic pollutants)	High (removes dyes, heavy metals, and pathogens)	Moderate (mainly used for sensing contaminants)	High (effective in removing dyes and heavy metals)

**Table 3:** TiO_2_ NPs: Synthesis, Characterization and Application by using different plant extract.

Sr. No.	Plants		Morphology	Size [nm]	Characterization	Application	References
1	*Kniphofia foliosa*	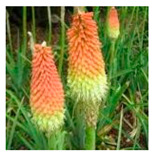	Spherical	----	UV–VIS, XRD, SEM TEM, TGA and DTA	Antibacterial against *Staphylococcus aureus*, *Escherichia coli*, *Klebsiella pneumonia*, and *Streptococcus pyogenes*	[90]
2	*Syzygium cumini*	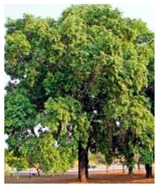	Irregular structure	18	UV–vis, HRTEM, TEM and XRD	Picric Acid Degradation and Anticancer activity	[91]
3	*Cassia fistula*	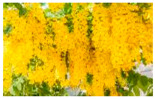	Spherical		UV–VIS, XRD, SEM, TEM and TGA	Antibacterial against *Escherichia coli* and *Staphylococcus aureus*	[92]
4	*Averrhoa bilimbi fruit*	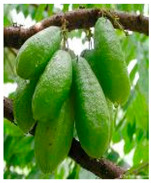	Spherical	1–8	EDX, FTIR, SEM, and TGA	Antibacterial against *S. aureus P. aeruginosa*, and *Candida albicans* fungi Degradation of methylene blue (MB)	[88]
5	*Carica papaya*	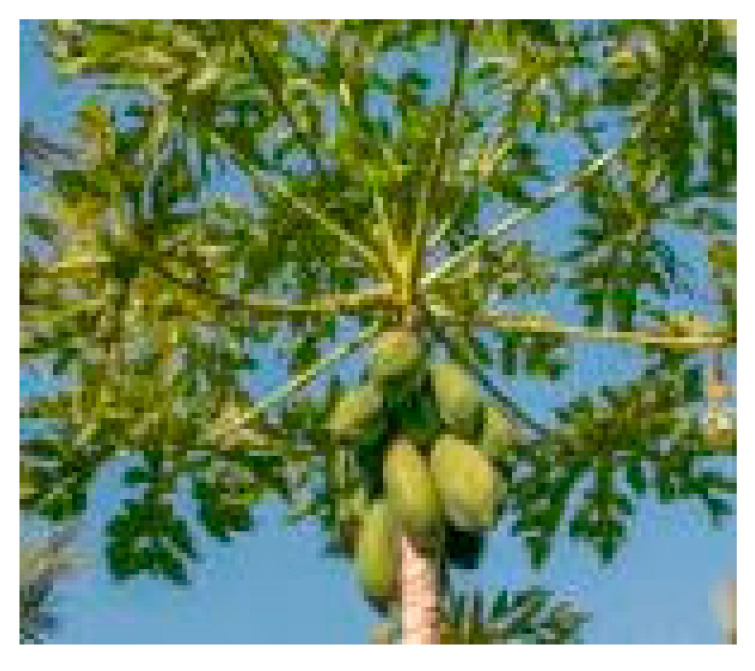	Spherical and cages like	15.6	UV–VIS, XRD, SEM and TEM	Degradation of dye	[84]
6	*Trianthema portulacastrum*	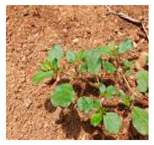	Porous crystallites with small round particles	10–12	XRD, FTIR, SEM and EDX	Antifungal against *U. tritici*	[76]
7	*Jatropha curcas* L.	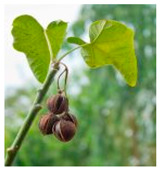	Spherical, tetragonal	13.0	UV–Visible, FESEM, EDS, FT-IR, XRD, DLS, BET and BJH	removal of chemical oxygen demand (COD) and chromium (Cr) from secondary treated TWW	[69]
8	*Azadirachta indica*	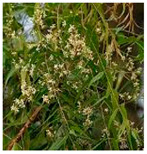	Spherical	124	XRD, SEM, TEM and FT-IR	*Escherichia coli*, *Staphylococcus aureus*, *Bacillus subtilis*, *Salmonella typhi* and *Klebsiella pneumonia*	[64]
9	*Alcea and Thyme*	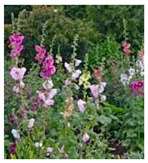	Irregularly shaped and spherical	10.1	XRD, FTIR, FESE and EDX	Degradation of methylene blue (MB)	[63]
10	*Jasmine Flowers*	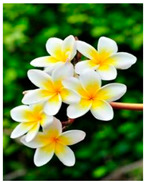	Spherical	31–42	XRD, UV−vis and SEM	Antibacterial against *Escherichia coli* and *Staphylococcus aureus* and Degradation of methylene blue (MB)	[61]
11	*Lagenaria siceraria* leaf	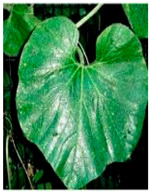	Irregular	303	XRD, FTIR, FESEM, EDX, HRTEM and UV−vis	photo-degradation of RG-19 dye	[60]
12	*Phyllanthus niruri* leaf	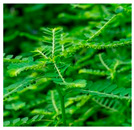	Crystalline	23	UV-DRS, EDX, FTIR, FESEM	Degradation of methylene blue (MB)	[59]
13	*Syzygium cumini*	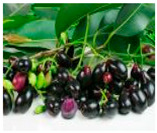	Spherical round shaped	22	HRSEM, HRTEM, EDS, FTIR, XRD, DLS and BET	lead removal from wastewater	[58]
14	*Tulbhagia violacela*	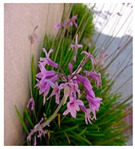	Rectangular and Irregular shaped	3.0–3.4	XRD, SEM, EDX, TEM, and UV−vis	antioxidant assay and Anticancer activity	[58]
15	*Commelina benghalensis*	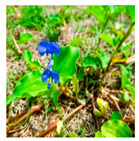	Spherical and agglomerated	30–200	XRD, SEM, EDX, BET, TGA and UV−vis	Degradation of methylene blue (MB) and SSX	[57]
16	*Wrightia tinctoria* leaf	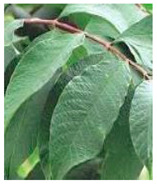	Spherical	22	FTIR, UV, XRD, HR-TEM and HR-SEM	Antifungal against *S. aureus*, *S. faecalis*, *E. coli*, *P. vulgaris*, *E. faecalis*, *P. aeruginosa*, *Y. enterocolitica*, *B. subtilis* and fungus *C. albicans*	[56]
17	*Nervilaaragona* leaf, *Ceaspinapulcherrima* flower, *Manihotesculante* plant extract	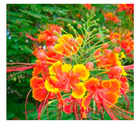	Spherical	15–28	XRD, FTIR, SEM, EDX, TEM and UV−vis	Antibacterial against *E. coli* and *S. aureus* and Degradation of methylene blue (MB)	[52]

## Data Availability

The data relevant to this work is presented in the manuscript.
